# Optimizing the Construction and Update Strategies for the Genomic Selection of Pig Reference and Candidate Populations in China

**DOI:** 10.3389/fgene.2022.938947

**Published:** 2022-06-08

**Authors:** Xia Wei, Tian Zhang, Ligang Wang, Longchao Zhang, Xinhua Hou, Hua Yan, Lixian Wang

**Affiliations:** ^1^ Key Laboratory of Farm Animal Genetic Resources and Germplasm Innovation of Ministry of Agriculture, Institute of Animal Science, Chinese Academy of Agricultural Sciences, Beijing, China; ^2^ State Key Laboratory Breeding Base of Dao-di Herbs, National Resource Center for Chinese Materia Medica, China Academy of Chinese Medical Sciences, Beijing, China

**Keywords:** genomic selection, optimizing strategies, reference population, candidate population, breeding scheme

## Abstract

Optimizing the construction and update strategies for reference and candidate populations is the basis of the application of genomic selection (GS). In this study, we first simulated1200-purebred-pigs population that have been popular in China for 20 generations to study the effects of different population sizes and the relationship between individuals of the reference and candidate populations. The results showed that the accuracy was positively correlated with the size of the reference population within the same generation (*r* = 0.9366, *p* < 0.05), while was negatively correlated with the number of generation intervals between the reference and candidate populations (*r* = −0.9267, *p* < 0.01). When the reference population accumulated more than seven generations, the accuracy began to decline. We then simulated the population structure of 1200 purebred pigs for five generations and studied the effects of different heritabilities (0.1, 0.3, and 0.5), genotyping proportions (20, 30, and 50%), and sex ratios on the accuracy of the genomic estimate breeding value (GEBV) and genetic progress. The results showed that if the proportion of genotyping individuals accounts for 20% of the candidate population, the traits with different heritabilities can be genotyped according to the sex ratio of 1:1male to female. If the proportion is 30% and the traits are of low heritability (0.1), the sex ratio of 1:1 male to female is the best. If the traits are of medium or high heritability, the male-to-female ratio is 1:1, 1:2, or 2:1, which may achieve higher genetic progress. If the genotyping proportion is up to 50%, for low heritability traits (0.1), the proportion of sows from all genotyping individuals should not be less than 25%, and for the medium and high heritability traits, the optimal choice for the male-to-female ratio is 1:1, which may obtain the greatest genetic progress. This study provides a reference for determining a construction and update plan for the reference population of breeding pigs.

## 1 Introduction

Genomic selection (GS), a method by which breeding values are predicted using genome-wide markers, has become more and more popular in livestock breeding (Goddard et al., 2016). In pigs, there are many studies that have reported the advantage of GS. Using pig 60K chips, Christensen et al. studied the daily gain and feed efficiency of Danish Duroc pigs and found that the genomic estimate breeding value (GEBV) is more accurate than traditional methods (Christensen et al., 2012). Uimari et al. performed GS on 86 Landrace pigs and 32 Large White pigs using a 60K chip for traits such as growth rate, feed conversion ratio, and carcass quality, and the results showed that the effective population size estimated by the genomic best linear unbiased prediction (GBLUP) and best linear unbiased prediction (BLUP) methods was almost indistinguishable, indicating that the genome selection technique has obvious advantages in the case of insufficient data volume (Uimari and Tapio, 2011). In the research of Howard et al., the single-step genomic best linear unbiased prediction has more accurate than BLUP (Howard et al., 2018). The findings of Marulanda et al. suggest that GS can greatly improve genetic progression (Marulanda et al., 2021).

There are many factors that affect the accuracy of genome selection, one of which is the size and composition of the reference population (Yang et al., 2020). The reference population consists of individuals with genotype information and observations of phenotypic values based on their own or offspring performance. In general, the accuracy of the GEBV will be higher when using a larger reference population (Dekkers et al., 2021). An optimally designed reference population is expected to maximize the accuracy of a candidate population (Isidro et al., 2015; Marulanda et al., 2021). In the research of Cleveand et al., the accuracy of the GEBV was related to the degree of kinship between the reference population and the candidate population, so parental information was used to predict the offspring with the highest accuracy (Cleveland et al., 2012). In other research, if the reference population was not updated, the accuracy of genomic breeding values decreased with increasing generations (Meuwissen et al., 2001; Pszczola et al., 2012a). Moreover, as data continue to accumulate, the estimation pressure continues to increase; in particular, the time spent on the inversion of the G matrix increases. Therefore, maintaining a suitable number of reference populations is particularly important for the application of GS in production practice.

Candidate populations are another important component of genomic selection. However, due to the limitation of research and development costs, different companies have different requirements for the determination of the proportion of candidate populations, and according to the characteristics of different traits, the genetic progress obtained by different genotyping schemes is also be inconsistent. The results of Danish’s pig research Centre showed that the greatest potential genetic progress can be obtained by preselecting 40% of the individuals for genotyping using the mean GEBV of the parents. In the research of Lillehammer et al., if two sibs of boars were tested simultaneously in each litter, the genetic progress of maternal traits was faster (Lillehammer et al., 2011). However, little research has reported the genotyping proportion and sex ratio of traits with different heritabilities in pigs.

In China, there are more than 3000 breeding farms, and the GS was first implemented in Wens group at 2012. At 2017, China national GS plan which consisted of about 30 farms was implemented, and then the reference population began to be constructed. However, systemic GS breeding scheme for most of the breeding farms is still lacked. In this study, based on the most popular population structure of pig farms in China, the effect of the reference population size and update strategy on the accuracy of genome selection was studied by simulating swine genome data. The effect of the genotyping amount and genotyping ratio of the candidate population on the accuracy of genome selection and genetic progress was also explored. According to the actual situation of breeding and production in breeding pig farms, this study provides a technical reference for the establishment of the application of GS technology in the genetic evaluation of breeding pigs.

## 2 Materials and Methods

### 2.1 Data Simulation

In this study, QMSim software (Sargolzaei and Schenkel, 2009) was used to simulate pedigree and genomic data. When simulating the reference population formation and update strategy, the selected medium heritability traits were set to 0.3. To research the candidate population, data for three different heritabilities (0.1, 0.3, and 0.5) were simulated.

#### 2.1.1 Historical Population

**FIGURE 1:**
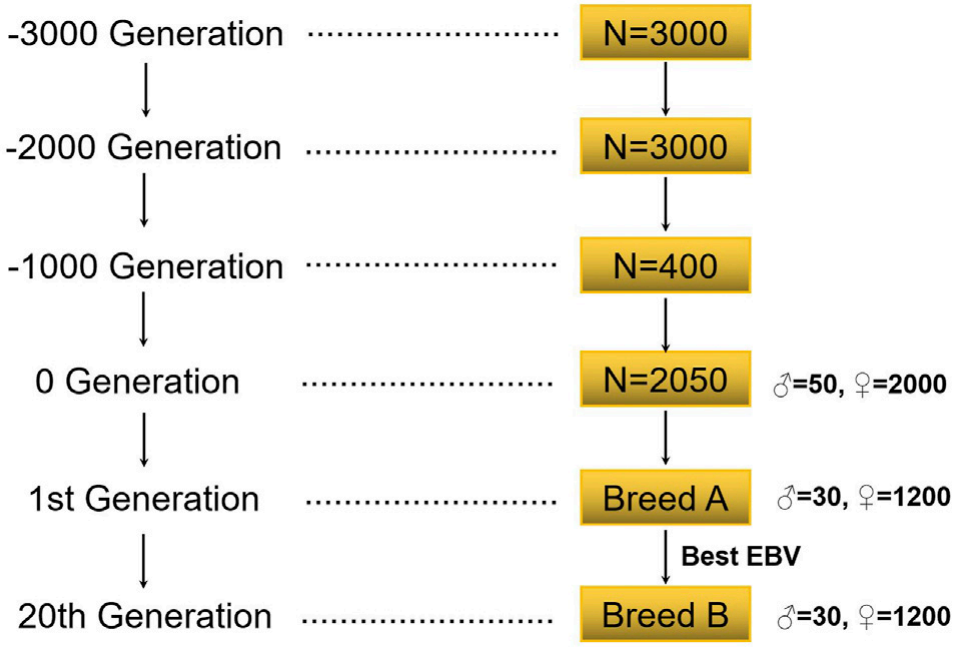
The flow of population simulation.

The simulation flow of this study is shown in Figure 1. This study first simulated the bottleneck effect of the population in the historical evolution process according to the Fisher–Wright population model (Wright, 1990). The initial population was 3000 heads, and 1000 generations were simulated. The population of the 1000th generation was 3000, with half being male and half female. After 1000 generations, the size of the population was gradually reduced to 400. The last generation obtained 2050 individuals (including 50 male individuals) by random mating. These individuals formed the basis of all selection schemes, and recent populations were selected on this basis.

#### 2.1.2 Recent Population

Simulation was carried out according to the group size of a general purebred pig farm in China (Song et al., 2017; Guan et al., 2021). In the population, there were 30 boars, 1200 sows, a male-to-female ratio of 1:40, 10 offspring per litter, and a male to female offspring ratio of 1:1; the annual renewal rate of the boars in the group was 100%, and the annual renewal rate of the sows was 40%. In each generation, individuals with a high EBV were selected and individuals with a low EBV were eliminated. When studying the reference population, 20 generations of data were simulated, and the mating method was random mating. In the research on the candidate population, five generations of data were simulated, and the mating method was also random mating (Table 1).

**TABLE 1:** Simulation group structure composition and update strategy.

Boars	Sows	Litter size	Test number		Refresh rate (%)	Refresh number	Raising rate (%)	Boar/Gilts	Fraction selected	
			BLUP	GBLUP					BLUP	GBLUP
30	1200	10	120	1200	100	30	80	37.5	0.016	0.016
30	1200	10	2400	1200	40	480	80	600	0.125	0.25

#### 2.1.3 Genome Parameters

According to the Porcine SNP50 BeadChip chip, which is commonly used in China, the simulated genome parameters were set as follows: The number of chromosomes was 18, the chromosome length was 100 M, the number of SNP markers on each chromosome was 2834, and the number of Single Nucleotide Polymorphisms (SNP); markers on the genome was 51,012. There were 17 QTLs on each chromosome, both markers and quantitative trait loci (QTLs) were biallelic, and SNPs and QTLs are evenly distributed on the chromosomes. The distribution of QTL effects follows a gamma distribution with ɑ = 0.4 and a scale function of 1.66, according to Hayes et al. (Hayes and Goddard, 2001).

### 2.2 Estimate Breeding Value Estimate Model

The EBV that was used to generate the index was estimated based on the animal model as outlined below:

y=Xb+Zg+e

where *y* is a vector of phenotypic observations, *b* is a vector of fixed effects, *u* is a vector of random additive genetic effects, *e* is a vector of random residuals, and *X* and *Z* are incidence matrices relating observations to the fixed and random additive genetic effects, respectively.

The GEBV was estimated using single-step GBLUP (ssGBLUP) model which could using information of both genotyped and non-genotyped phenotype information. The ssGBLUP has the same model as EBV estimated, except vector g is assumed to follow a normal distribution N (0, Hσ_g_^2^), as described in previous study (Song et al., 2018).

### 2.3 Estimation Accuracy

The correlation between the true breeding value (TBV) and the EBV on the selection candidates were calculated as accuracy.

### 2.4 Estimation of the Breeding Values of the Reference Populations

In-house R scripts were used to randomly select one male and one female from each litter of each generation for phenotype calculation. The pedigree data were the pedigree of all individuals from the initial generation to the 20th generation. Using the DMUAI module in the DMU software, parameter estimation was performed for each group of generations, repeated three times, and the average value was used as the parameter for estimating the GEBV. The composition of each generation and the composition of the pedigree data are shown in Supplementary Table S1. In this study, the reference population was constructed in four ways: 1) randomly selecting one male and one female (20%), two males and two females (40%), three males and three females (60%), four males and four females (80%), five males and five females (100%),respectively, in the 19th generation; 2) randomly selecting one male and one female from each litter of each generation from the 10th to the 19th generation; 3) using a combination of different generations of the second case, and this combination is shown in Supplementary Table S2; 4) combining the first and third case populations to compare the differences in the accuracy of the distribution of the same number of reference populations in one generation and in different generations. In the reference population, all of the animals were with both phenotypes and genotypes. One male and one female were randomly selected from each litter of the 20th generation, with only genotype information and no phenotype information for random sampling with R scripts.

### 2.5 Estimation of the Breeding Values of the Candidate Populations

Using the pedigree data of all five generations and the phenotype data of one male and two females randomly selected from each litter of the first four generations, the parameters were estimated using the DMUAI module in the DMU software, repeated three times, and the mean was calculated as the parameters for GEBV and EBV estimation.

From the first four generations of individuals, one male and one female per litter were selected as the reference population. Selection of the candidate population set different schemes according to the determination and gender ratios. When the genotyping ratio was 20% of the candidate population, the selection ratios were 2:0, 1:1, and 0:2, respectively, that is, two boars, one male and one female, or two sows were selected from each litter. When the genotyping ratio was 30% of the candidate population, the ratio of male to female was selected as 3:0, 2:1, 1:1, 1:2, and 0:3. When the genotyping ratio was 50% of the candidate population, the male-to-female ratio was selected as 5:0, 4:1, 3:2, 1:1, 2: 3, 4:1, and 0:5. The GMATIX module in the DMU software was used to build the G matrix, while the DMU5 module was used to estimate the EBV, and the “Sing-step” method ssGBLUP was used to estimate the GEBV. The breeding rate of general breeding pigs in actual production is 80%, and 200 sows and 38 boars were selected from the offspring each year for updating the basic group.

### 2.6 Calculation of Genetic Progress

In the population update, the update rate of boars was 100% and sows was 40%. However, since this study only involved one generation, for the convenience of calculation, it was assumed that the generation interval of the population was 1. According to the genetic gain formula:

$$\Delta G_{t}=\Delta G/L=\left(\sigma_{A}*i*r_{AI}\right)/L$$

where*ΔG*_*t*_ is the annual genetic gain, *ΔG* is the total genetic gain, *L* is the generation internal, *σ*_*A*_ is the additive genetic variation, *i* is the selection intensity, and*r*_*AI*_ is the estimation accuracy.

Suppose that L = 1, then the final formula is:

ΔGt=ΔG=σA∗i∗rAI

## 3 Results

### 3.1 Genetic Parameter Estimation

**FIGURE 2:**
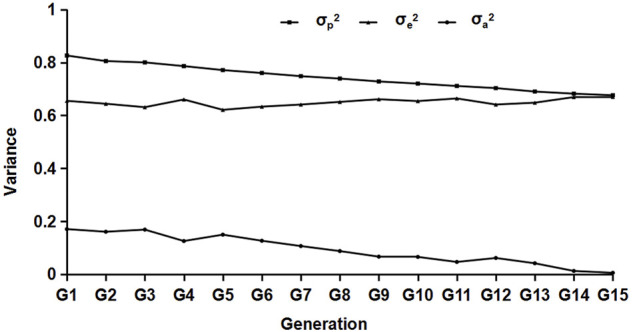
Results of the variance component estimation in different generations. σ_p_^2^,σ_a_^2^, and σ_e_^2^ represent the phenotypic, residual, and genetic variances, respectively.

Variance estimation was performed on the data of different generations, and the results are shown in Figure 2. From the results, we can see that with the increase in generations, the composition of each variance group decreased generation by generation, and the genetic variance decreased significantly.

As shown in Table 2, with the increase in heritability, the total variance group gradually decreased; when the heritability was 0.1 and 0.5, the residual variance was slightly higher than the theoretical value, but the genetic variance was lower than the theoretical value.

**TABLE 2:** Genetic parameter estimation of the different heritabilities in generation G15.

Heritabilities	0.1	0.3	0.5	0.8
σ_a_	0.088 ± 0.007	0.295 ± 0.016	0.457 ± 0.039	0.748 ± 0.052
σ_e_	0.921 ± 0.048	0.682 ± 0.033	0.513 ± 0.017	0.190 ± 0.009
σ_p_	1.09 ± 0.039	0.977 ± 0.055	0.970 ± 0.052	0.938 ± 0.047

Note: σ_p_^2^, σ_a_^2^, and σ_e_^2^ represents phenotypic variance, residual variance and genetic variance, respectively.

### 3.2 Accuracy of Genomic Estimate Breeding Value Estimation With Different Genotyping Proportions of the Reference Population in the Same Generation and in Different Generations

**FIGURE 3:**
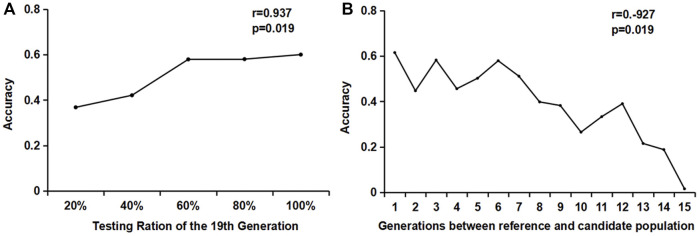
Estimation accuracy of the influence of different genotyping numbers and generations. **(A)** Changes in the accuracy of different reference populations in the same generation; **(B)** Effects of different reference population generations on the accuracy of the estimated breeding values.

It can be seen from Figure 3A that the accuracy of the GEBV estimation increased significantly with the increase in the genotyping proportion of the reference population when the candidate populations were the same. When the reference population genotyping increased from 40 to 60%, the accuracy value increased from 0.4171 to 0.5737, with an increase of 35%. When the reference population genotyping ratio increased from 60 to 100%, the estimated accuracy of the GEBV increased slower, with an increase of 7% (0.5718–0.6081).

When studying the accuracy of estimating the GEBV of different generations, the reference population consisted of individuals from a single generation. One male and one female were randomly selected from each litter of the 20th generation to form a candidate population. The genotype was detected, but no phenotype was recorded. One male and one female were selected from each litter of each generation from the 5th to the 19th generation to form 15 groups (1–15) of reference populations, with approximately 2400 individuals in each group. The GEBV accuracy of the 20th generation candidate population by reference populations of different generations is shown in Figure 3B. It can be seen from the figure that the GEBV accuracy increased as the generations between the reference population and the candidate populations became closer. The accuracy estimated using the 19th generation individuals as the reference population was significantly higher than that of the other generations. The correlation coefficient between the generations and the accuracy was −0.927, and the *p*-value was less than 0.01, showing a very significant correlation.

### 3.3 Accuracy of Estimating the Genomic Estimate Breeding Value in Reference Populations of Different Generations

**FIGURE 4:**
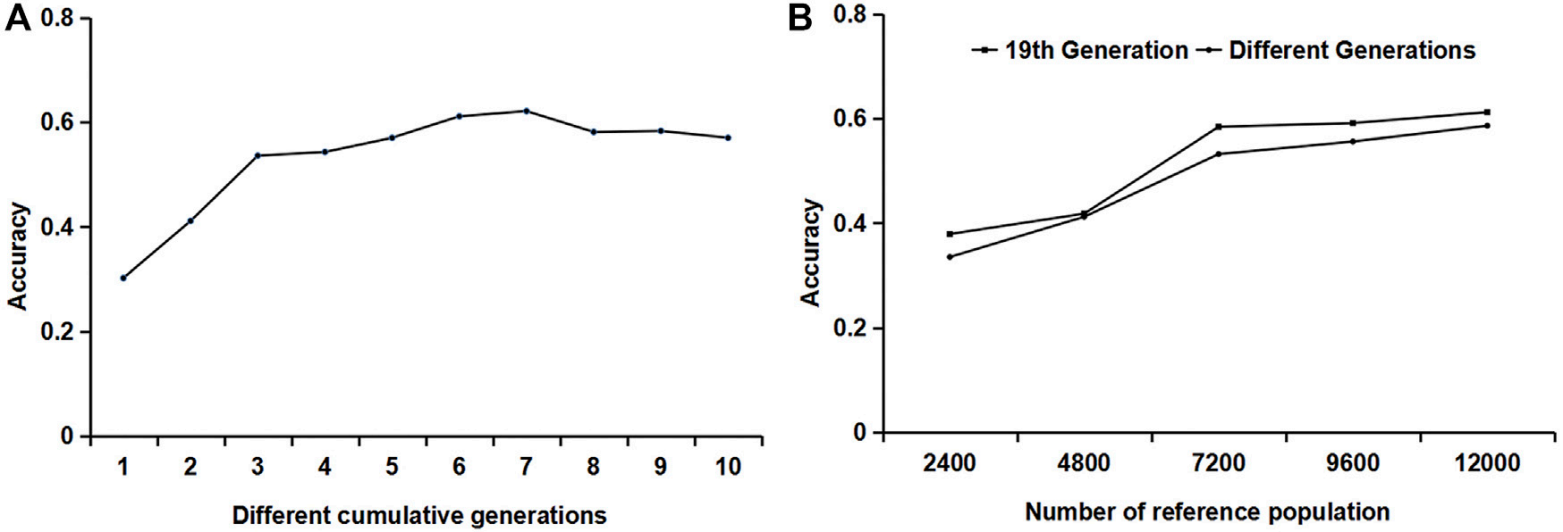
The influence of different generations and different relationships of the reference populations on accuracy. **(A)** The influence of reference populations from different generations on accuracy. **(B)** The influence of the relationship between reference individuals on accuracy.

A combination of different generations was used as the reference population. One male and one female were randomly selected from each generation and litter for genotype and phenotype detection. The variation trend of the accuracy is shown in Figure 4A. When the 19th, 18th–19th, and 17th–19th generations were used as the reference populations, the accuracy of the first three generations accumulated by the reference population increased rapidly. From the accumulation of the third generation to the seventh generation, the increase in accuracy slowed down. When the reference population accumulated over seven generations, the accuracy began to decline. Therefore, it is recommended that the reference population should be updated when the cumulative generation exceeds seven generations.

### 3.4 The Effect of Kinship Between Reference Populations on the Accuracy of the Genomic Estimate Breeding Value

For reference populations in the same generation, different numbers of individuals per litter were selected: one male and one female, two males and two females, three males and three females, four males and four females, and five males and five females, respectively. For the reference populations from different generations, one male and one female combination were randomly selected from each generation and each litter to form a reference population. In one generation or different generations, the accuracy increased with the increase in the number of reference populations; when the number of reference populations was constant, such as the reference population of 7200 heads, the accuracy of individuals in the reference population from the same generation was slightly lower.

### 3.5 Selection Accuracy and Genetic Progress of the Genomic Estimate Breeding Value Estimated by Different Candidate Population Genotyping Proportions

**FIGURE 5:**
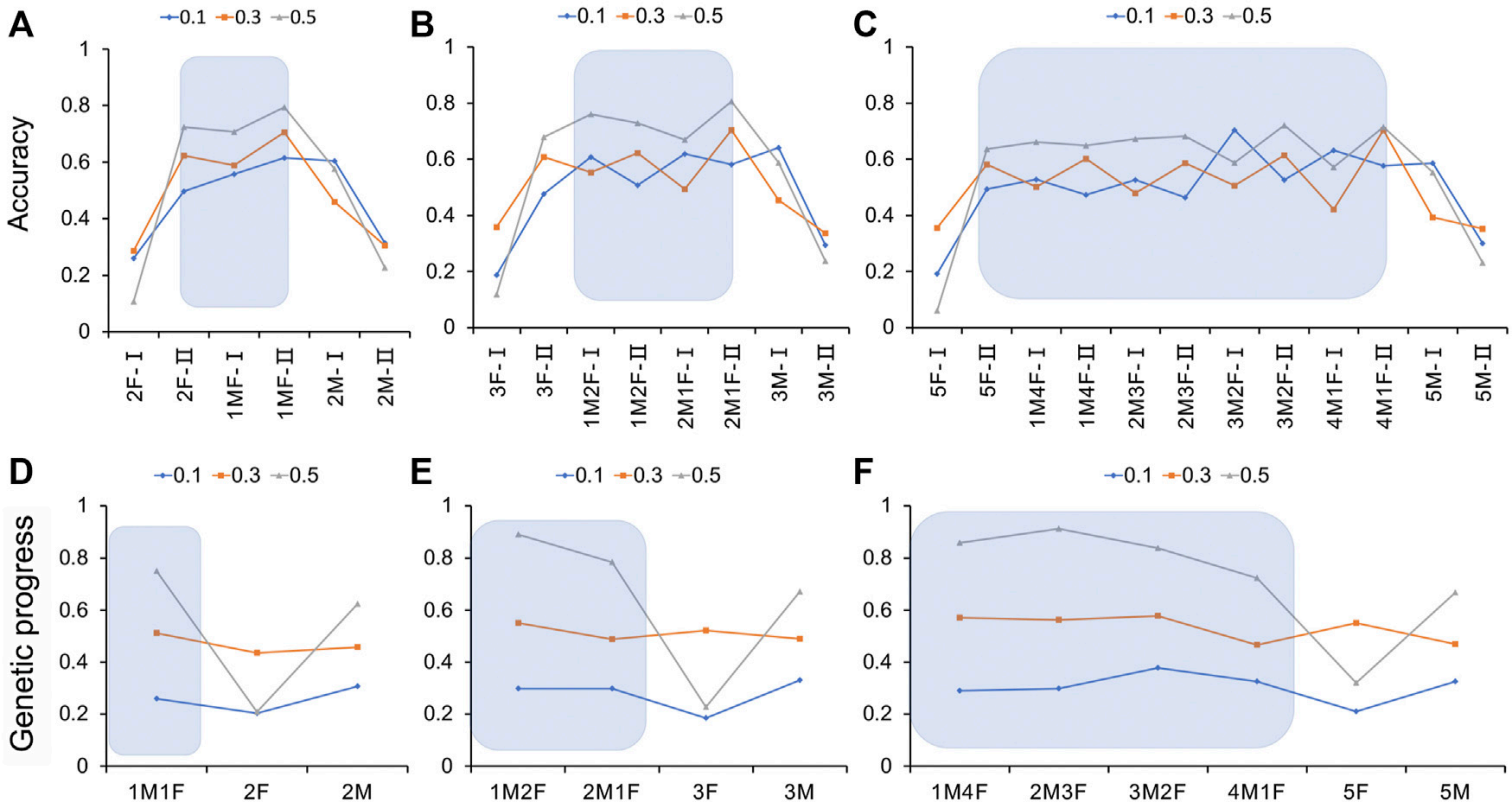
Estimation accuracy and genetic progress when the genotyping ratio was 20, 30, and 50% of the candidate population for different heritability traits. **(A**–**C)** Estimation accuracy when the genotyping ratio was 20, 30, and 50% of the candidate population for different heritability traits; **(D**–**F)** Genetic progress when the genotyping ratio was 20, 30, and 50% of the candidate population for different heritability traits. M, male; F, female; I, boar; II, gilt.

It can be seen from the results (Figure 5A and Supplementary Table S3) that for the genome selection of 20% genotyping ratio, when the genotyping protocol was a 1:1 ratio of males to females, the selection accuracy of the sows was the highest at 0.615, 0.706, and 0.795, respectively, for heritability of 0.1, 0.3, and 0.5. With the increase in the heritability of the trait, the selection accuracy also increased. If only one sex was genotyped individually, the selection accuracy of the other sex was significantly reduced. When the male to female genotyping ratio was 1:1, there was a significant advantage in the genetic progress obtained by the traits of different heritabilities (Figure 5D and Supplementary Table S3). When the heritability was 0.1 or 0.3, the sex ratio had little effect. When the heritability reached 0.5, the genetic progress was significantly higher in the male to female 1:1 and 2:0 genotyping groups than in the 0:2 genotyping group.

For 30% of the genome selection of the genotyping ratio, when the trait heritability was 0.1 and the genotyping male-to-female ratio was 1:1, the selection accuracy of the boars was the highest, at 0.708. When the genotyping ratio of males to females was 2:1, the selection accuracy of the sows was the highest, at 0.704 and 0.806, respectively, in the medium and high heritability groups. If only one sex was genotyped, the selection accuracy of the other sex was significantly lower (Figure 5B and Supplementary Table S4). When the genotyping ratio of males to females was 1:1, the genetic progress obtained for heritability traits of 0.1 was 0.339, and the difference was significant [*p* < 0.05 higher than the other groups (the genotyping ratio was not 1:1)]. When the heritability of the trait was 0.3 or 0.5, the genetic progress of the male to female genotyping ratio 1:2 of was the highest (Figure 5E and Supplementary Table S4).

As shown in Figure 5C and Supplementary Table S5, it can be seen that for 50% of the genome selection of the genotyping ratio, the selection accuracy increased with the heritability. When the trait heritability was 0.1 and the genotyping ratio of males to females was 3:2, the highest accuracy was 0.704. When the heritability was medium and high, if the male to female genotyping ratio was 4:1, the selection accuracy of the sows was the highest. If only one sex was genotyped, the selection accuracy of the other sex was significantly reduced. When the heritability of the trait was 0.1, the maximum genetic progress could be obtained when the male-to-female ratio of the individuals for genotype determination was 3:2. When the heritability of the trait was 0.3, the lowest genetic progress was obtained when the genotyping ratio of males to females was 4:1. When the heritability of the trait was 0.5 and the ratios of the genotyped individuals were 1:4, 1:1, 2:3, and 3:2, the genetic progress values were 0.859, 0.859, 0.912, and 0.912, respectively (Figure 5F and Supplementary Table S5).

### 3.6 Influence of Different Genotyping Ratios and Different Heritabilities on the Accuracy of Estimating the Genomic Estimate Breeding Value

**FIGURE 6:**
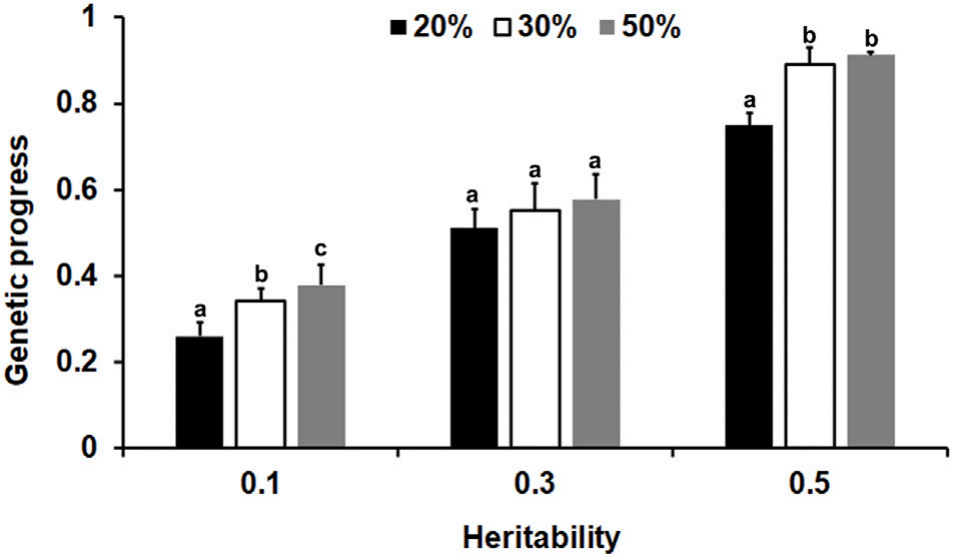
The influence of the genotype ratio and heredity on the genetic progress of selection. Different lowercase letters above the bars represent significant differences (*p* < 0.05).

From the results of the genetic progress in this study (Supplementary Table S3), it can be seen that for the different heritability traits, when the offspring of 1200 basic groups were measured by 20%, the genetic progress of the group improved by 53–100% compared to the traditional BLUP method for phenotypic genotyping of one male and two females. We then chose the optimal sex proportion described above to determine the optimal genotyping proportion. As shown in Figure 6, generally, the genetic progress increased when the genotyping proportion of the offspring increased. For the lower heritability traits, the genetic progress of a 50% genotyping ratio was the highest, and the difference between the 30 and 50% genotyping ratios was significant (*p* < 0.05). Moreover, for the medium and high heritability traits, when the genotyping ratio reached 30%, the accuracy was reasonable (*p* < 0.05).

## 4 Discussion

GS techniques have been widely used in livestock and plant breeding due to their ability to achieve higher estimation accuracy and greater genetic progress (Iqbal et al., 2019). The accuracy of genomic estimates of breeding values is influenced by marker density (Meuwissen et al., 2001), number of reference populations (Daetwyler et al., 2008; Hayes et al., 2009a), size and number of QTL effects (Kizilkaya et al., 2010; Daetwyler et al., 2012), and the degree of linkage disequilibrium between markers and QTLs (Habier et al., 2010). The establishment of a reference population is one of the primary tasks and core components of the application of GS technology in livestock and poultry breeding and has an important impact on the accuracy of the estimated breeding value of the genome (Pszczola and Calus, 2016). In general, the larger the reference population, the higher the accuracy of the genome’s estimated breeding values (Hayes et al., 2009b; Goddard et al., 2011). The results of this study, based on simulating the population structure of purebred pigs, also show that the accuracy of the genome-estimated breeding value is almost linearly related to the size of the reference population, and is significantly positively correlated with the number of reference populations, whereby the correlation coefficient reached 0.9366 (*p* < 0.05).

The optimal design of reference populations could maximize the accuracy of estimated breeding values for a given population’s genome (Clark et al., 2012; Rincent et al., 2012). Simulation studies have shown that the farther the genetic relationship between the reference population and the closer the genetic relationship to the candidate population, the higher the accuracy of the estimated breeding value of the genome (Pszczola et al., 2012a; Pszczola et al., 2012b; Isidro et al., 2015; Wu et al., 2015). If the reference population is not updated, both the accuracy and reliability of the genome’s estimated breeding values will decrease as the distance between the reference population and the candidate population increases (Calus and Veerkamp, 2011; Wolc et al., 2011; Lillehammer et al., 2016; Castro Dias Cuyabano et al., 2019). Our study simulated the group structure of pigs, and the reference population was composed of 10 generations. Within seven generations, the increasing trend of accuracy is consistent with the results of Weng et al. (Weng et al., 2016). After the generation exceeded seven generations, the accuracy decreased. There are some differences between this study and the study of Weng et al., for which there may be two reasons: First, the number of generations simulated in this study was relatively large, and second, the two studies involved different animal species. Therefore, in the implementation of genome selection on pigs, the accumulation of reference populations with generations is not the best. On the one hand, as genotyping data continue to accumulate, the size of the reference population is also an important factor limiting the efficiency of GS techniques. The computing and storage capacity will be limited with the increase in the number of reference populations. On the other hand, with the increase in the cumulative generation of candidate populations, the number of individuals with distant genetic relationships between the candidate and reference populations increases. The original linkage disequilibrium between SNPs and QTLs will weaken, and new linkage disequilibria may also be established. The results of our study show that the accuracy of genome selection decreased significantly with increasing generations between the reference and candidate populations. Therefore, during the implementation of GS, it is crucial to keep the reference population information updated to maintain the accuracy of GS. Therefore, the SNP effect estimated by a reference population that is far from the candidate population will be biased, and the accuracy of the GEBV estimated by the SNP effect will also decrease. Moreover, our results indicate that the accuracy of genome selection does not increase infinitely with the accumulation of reference groups of different generations, but shows a trend of first improving and then lowering.

During the implementation of genome selection technology, if the cost of gene chips does not drop significantly, it is currently impossible for domestic breeding livestock and poultry enterprises to genotype all individuals in the whole herd (Henryon et al., 2014; Meuwissen et al., 2016; Wolc et al., 2016). Strategies for genotyping selection candidates should be explored to maximize the economic benefit of GS (Granleese et al., 2019). This study selected individuals for genotyping on a litter-based unit in the candidate population to ensure that the selected individuals are representative. In the results of Lillehammer et al., when three boars were measured in each litter and all sows were genotyped, the genetic progress for reproductive traits was the largest, when 50% of the offspring were genotyped without testing for boars, the genetic progress was slightly decreased but was cost-effective (Lillehammer et al., 2011). The results of this study showed that when the proportion of genotyped individuals accounted for 20% of the candidate population, the traits of different heritabilities could be genotyped according to a male-to-female sex ratio of 1:1. If the genotyped individuals account for 30% of the candidate population, for low heritability (0.1) traits, it is recommended that the ratio of male to female for genotyping should be 1:1. Higher genetic progress can be obtained when the ratio of males to females is 1:1, 1:2, and 2:1 for medium and high heritability traits. When the genotyping ratio reaches 50% of the candidate population, the ratio of sows for low heritability traits (0.1) should not be less than 25%, and the ratio of male to female genotyping for medium and high heritability traits should be 1:1 to achieve the best genetic progress.

In the 2010 annual report of the DanBred Swine Genetics Research Center (Pig Research Centre, PRC), issues such as the genotyping ratio of the candidate population and how to select genotyped individuals were discussed. The EBV value was estimated by the BLUP method, and the optimal 40% individuals was selected for genotyping according to the EBV value. When the male-to-female ratio of genotyping was 3:1, the genetic progress equivalent to the genotyping of the whole group could be obtained (Annual Report of Centre Danish Pig Research). The design ideas of this study are quite different from those of DanBred. DanBred implements genome selection after performance measurement, while this study fully considered the problems of performance genotyping in China and the cost of breeding and management of breeding pigs. We hope to use GS technology to pre-select piglets at birth to reduce the feeding volume of breeding pigs, especially the feeding volume of boars, and reduce the cost in the process of breeding pigs. The scheme of DanBred is a good strategy for reducing the cost of genotyping, but because the parents of siblings have the same EBVs, 40% of the individuals selected according to the average EBVs of parents occupy a large proportion of siblings. Continued breeding according to this scheme will lose some of them. Genetic variation affects the genetic progression of a population. Judging from the current research, various schemes have their own advantages and disadvantages, and further research and exploration are needed to establish a complete set of pig genome selection schemes.

## 5 Conclusion

This study suggests that when initially constructing a reference population, the number of reference population should be large enough, and individuals with the closest kinship to the candidate population should be selected. In addition, the replacement of new and old generations of reference populations can be carried out from the fourth generation at the earliest at the seventh generation at the latest. For the selection of lower heritability traits, when the candidate individual genotyping ratio reach 50%, the genetic progress was acceptable, while for the medium and high heritability traits, when the genotyping ratio reached 30%, the genetic progress was acceptable. If the proportion of genotyped individuals accounts for 20% of the candidate population, the male-to-female sex ratio of 1:1 is preferred for all heritability traits. If genotyped individuals account for 30% of the candidate population, it is recommended that the male-to-female ratio of low heritability (0.1) traits be genotyped at 1:1, with male-to-female ratios for medium and high heritability traits at 1:1, 1:2, and 2:1. If the genotyping ratio reaches 50% of the candidate population, the proportion of sows with low heritability traits (0.1) cannot be less than 25%. In conclusion, this study provides a detailed reference for the establishment of the application of GS technology in the genetic evaluation of breeding pigs.

## Data Availability

The original contributions presented in the study are included in the article/Supplementary Material, further inquiries can be directed to the corresponding authors.

## Abbreviations

BLUP, best linear unbiased prediction; EBV, estimate breeding value; GEBV, genomic estimate breeding value; GBLUP, genomic best linear unbiased prediction; GS, Genomic selection; QTLs, quantitative trait loci; SNP, Single Nucleotide Polymorphisms; TBV, true breeding value.
