# Supplementary material for: Optimizing the Construction and Update Strategies for the Genomic Selection of Pig Reference and Candidate Populations in China
Source: Front Genet. 2022 Jun 8;13:938947. doi: 10.3389/fgene.2022.938947 (PMC9213789; doi:10.3389/fgene.2022.938947)
Supplement: Supplementary file 1 [file DataSheet1.docx]

**Table S1. The phenotypes and pedigree data for each group**

| Group | Construction generation | Number of individuals with Phenotype | pedigree |
| --- | --- | --- | --- |
| G1 | 5-19 | 36000 | 192000 |
| G2 | 6-19 | 33600 | 180000 |
| G3 | 7-19 | 31200 | 178000 |
| G4 | 8-19 | 28800 | 156000 |
| G5 | 9-19 | 26400 | 144000 |
| G6 | 10-19 | 24000 | 132000 |
| G7 | 11-19 | 21600 | 120000 |
| G8 | 12-19 | 19200 | 108000 |
| G9 | 12-19 | 16800 | 96000 |
| G10 | 14-19 | 14400 | 84000 |
| G11 | 15-19 | 12000 | 72000 |
| G12 | 16-19 | 9600 | 60000 |
| G13 | 17-19 | 4200 | 48000 |
| G14 | 18-19 | 4800 | 36000 |
| G15 | 19 | 2400 | 24000 |

**Table S2. The number of reference population by different generations**

| Group | Generation | Number of Individuals |
| --- | --- | --- |
| g1 | 19 | 2400 |
| g2 | 18-19 | 4800 |
| g3 | 17-19 | 7200 |
| g4 | 16-19 | 9600 |
| g5 | 15-19 | 12000 |
| g6 | 14-19 | 14400 |
| g7 | 12-19 | 16800 |
| g8 | 12-19 | 19200 |
| g9 | 11-19 | 21600 |
| g10 | 10-19 | 24000 |

**Table S3. Comparison between the accuracy and genetic progress of GEBV estimation in different testing schemes of the candidate population and the BLUP method when the testing proportion of the total candidate population is 20%**

|  | Group | Heritability |  |  |
| --- | --- | --- | --- | --- |
|  |  | 0.1 | 0.3 | 0.5 |
| Accuracy | 2F-Ⅰ | 0.260±0.093^C^ | 0.287±0.073^E^ | 0.108±0.037^F^ |
|  | 2F-Ⅱ | 0.497±0.005^B^ | 0.623±0.017^AB^ | 0.725±0.011^AB^ |
|  | 1MF-Ⅰ | 0.558±0.095^AB^ | 0.589±0.072^B^ | 0.707±0.04^B^ |
|  | 1MF-Ⅱ | 0.615±0.013^A^ | 0.706±0.02^A^ | 0.795±0.01^A^ |
|  | 2M-Ⅰ | 0.604±0.126^AB^ | 0.460±0.039^CD^ | 0.577±0.019^C^ |
|  | 2M-Ⅱ | 0.314±0.019^C^ | 0.306±0.018^E^ | 0.227±0.019^E^ |
|  | EBV-Ⅰ | 0.320±0.029^F^ | 0.376±0.096^G^ | 0.450±0.100^F^ |
|  | EBV-Ⅱ | 0.230±0.032^FG^ | 0.472±0.014^CD^ | 0.464±0.018^F^ |
| Genetic progress | 2F | 0.203±0.04^BC^ | 0.436±0.059^AB^ | 0.208±0.032^C^ |
|  | 2M | 0.306±0.047^A^ | 0.458±0.029^AB^ | 0.623±0.027^B^ |
|  | MF | 0.259±0.033^AB^ | 0.511±0.043^A^ | 0.75±0.028^A^ |
|  | EBV | 0.15±0.015^C^ | 0.391±0.054^B^ | 0.541±0.081^B^ |

Note: Ⅰ is male and Ⅱ is female. Different capital letters indicate significant differences in genetic progression between different assay protocols (p<0.05).

**Table S4. Comparison between the accuracy and genetic progress of GEBV estimation in different testing schemes of the candidate population and the BLUP method when the testing proportion of the total candidate population is 30%**

|  | group | Heritability |  | |  | |  |
| --- | --- | --- | --- | --- | --- | --- | --- |
|  |  | 0.1 | | 0.3 | | 0.5 | |
| Accuracy | 3F-Ⅰ | 0.187±0.052^G^ | | 0.358±0.099^EF^ | | 0.119±0.045^H^ | |
|  | 3F-Ⅱ | 0.477±0.017^D^ | | 0.608±0.019^AB^ | | 0.678±0.017^C^ | |
|  | 1M2F-Ⅰ | 0.608±0.085^B^ | | 0.553±0.097^BC^ | | 0.761±0.041^AB^ | |
|  | 1M2F-Ⅱ | 0.508±0.013^D^ | | 0.622±0.015^AB^ | | 0.729±0.016^BC^ | |
|  | 1.5MF-Ⅰ | 0.708±0.057^A^ | | 0.463±0.042^CDE^ | | 0.599±0.049^DE^ | |
|  | 1.5MF-Ⅱ | 0.521±0.025^CD^ | | 0.631±0.005^AB^ | | 0.720±0.008^BC^ | |
|  | 2M1F-Ⅰ | 0.619±0.059^B^ | | 0.493±0.139^C^ | | 0.670±0.040^CD^ | |
|  | 2M1F-Ⅱ | 0.581±0.023^BC^ | | 0.704±0.024^A^ | | 0.806±0.008^A^ | |
|  | 3M-Ⅰ | 0.641±0.043^AB^ | | 0.455±0.039^CDE^ | | 0.588±0.046^E^ | |
|  | 3M-Ⅱ | 0.295±0.009^EF^ | | 0.336±0.014^F^ | | 0.237±0.022^G^ | |
|  | EBV-Ⅰ | 0.320±0.029^E^ | | 0.376±0.096^EF^ | | 0.450±0.100^F^ | |
|  | EBV-Ⅱ | 0.230±0.032^C^ | | 0.472±0.014^C^ | | 0.464±0.018^D^ | |
| Genetic progress | 1.5MF | 0.339±0.023^A^ | | 0.492±0.028^AB^ | | 0.758±0.038^BC^ | |
|  | 1M2F | 0.298±0.028^B^ | | 0.551±0.064^A^ | | 0.891±0.038^A^ | |
|  | 2M1F | 0.299±0.02^B^ | | 0.487±0.092^AB^ | | 0.784±0.036^B^ | |
|  | 3F | 0.184±0.018^C^ | | 0.521±0.072^A^ | | 0.227±0.051^E^ | |
|  | 3M | 0.33±0.015^AB^ | | 0.489±0.034^AB^ | | 0.671±0.037^C^ | |

Note: I: represents the male gender; II: represents the female gender. Different capital letters indicate significant differences (p < 0.05) between the accuracies of different assay protocols in estimating breeding values.

**Table S5. Comparison between the accuracy and genetic progress of GEBV estimation in different testing schemes of the candidate population and the BLUP method when the testing proportion of the total candidate population is 50%**

|  | Group | Heritability |  |  | |
| --- | --- | --- | --- | --- | --- |
|  |  | 0.1 | 0.3 | 0.5 |  |
| Accuracy | 5F-Ⅰ | 0.192±0.017^G^ | 0.356±0.051^G^ | 0.06±0.076^H^ |  |
|  | 5F-Ⅱ | 0.493±0.008^CDE^ | 0.582±0.009^BC^ | 0.637±0.003^ABCD^ |  |
|  | 1M4F-Ⅰ | 0.528±0.158^CDE^ | 0.501±0.111^D^ | 0.661±0.095^ABC^ |  |
|  | 1M4F-Ⅱ | 0.473±0.016^E^ | 0.602±0.013^B^ | 0.649±0.006^ABCD^ |  |
|  | 2M3F-Ⅰ | 0.526±0.127^CDE^ | 0.479±0.082^DE^ | 0.672±0.1^ABC^ |  |
|  | 2M3F-Ⅱ | 0.464±0.004^E^ | 0.586±0.006^B^ | 0.681±0.015^AB^ |  |
|  | 2.5MF-Ⅰ | 0.635±0.02^AB^ | 0.459±0.042^DEF^ | 0.601±0.008^BCD^ |  |
|  | 2.5MF-Ⅱ | 0.484±0.001^DE^ | 0.608±0.007^B^ | 0.686±0.006^AB^ |  |
|  | 3M2F-Ⅰ | 0.704±0.098^A^ | 0.506±0.006^CD^ | 0.587±0.034^BCD^ |  |
|  | 3M2F-Ⅱ | 0.527±0.025^CDE^ | 0.614±0.003^B^ | 0.721±0.009^A^ |  |
|  | 4M1F-Ⅰ | 0.632±0.032^AB^ | 0.422±0.011^EFG^ | 0.572±0.014^CD^ |  |
|  | 4M1F-Ⅱ | 0.576±0.02^BCD^ | 0.704±0.021^A^ | 0.715±0.15^A^ |  |
|  | 5M-Ⅰ | 0.586±0.029^BC^ | 0.393±0.032^GF^ | 0.553±0.015^DE^ |  |
|  | 5M-Ⅱ | 0.301±0.017^FG^ | 0.353±0.016^G^ | 0.231±0.003^G^ |  |
|  | EBV-Ⅰ | 0.32±0.029^C^ | 0.376±0.096^DE^ | 0.45±0.1^D^ |  |
|  | EBV-Ⅱ | 0.23±0.032^FG^ | 0.472±0.014^DE^ | 0.464±0.018^EF^ |  |
| Genetic progress | 1M4F | 0.29±0.053^C^ | 0.57±0.064^A^ | 0.859±0.073^A^ |  |
|  | 2.5MF | 0.349±0.008^AB^ | 0.559±0.029^A^ | 0.859±0.01^A^ |  |
|  | 2M3F | 0.298±0.047^BC^ | 0.562±0.057^A^ | 0.912±0.08^A^ |  |
|  | 3M2F | 0.378±0.042^A^ | 0.578±0.004^A^ | 0.838±0.03^A^ |  |
|  | 4M1F | 0.325±0.014^ABC^ | 0.466±0.006^B^ | 0.723±0.031^B^ |  |
|  | 5F | 0.21±0.007^D^ | 0.551±0.039^A^ | 0.321±0.074^C^ |  |
|  | 5M | 0.325±0.009^ABC^ | 0.47±0.021^B^ | 0.667±0.015^B^ |  |

Note: I: represents the male gender; II: represents the female gender. Different capital letters indicate significant differences (p < 0.05) between the accuracies of different assay protocols in estimating breeding values.
